# The Anti-Genotoxic Activity of Wastewaters Produced after Water-Steam Distillation of Bulgarian *Rosa damascena* Mill. and *Rosa alba* L. Essential Oils

**DOI:** 10.3390/life12030455

**Published:** 2022-03-19

**Authors:** Svetla Gateva, Gabriele Jovtchev, Tsveta Angelova, Ana Dobreva, Milka Mileva

**Affiliations:** 1 Institute of Biodiversity and Ecosystem Research, Bulgarian Academy of Sciences, 1113 Sofia, Bulgaria; spetkova2002@yahoo.co.uk (S.G.); gjovtchev@yahoo.de (G.J.); angelova_ts@abv.bg (T.A.); 2 Institute for Roses and Aromatic Plants, Agricultural Academy, 49 Osvobojdenie Blvd., 6100 Kazanlak, Bulgaria; 3 The Stephan Angeloff Institute of Microbiology, Bulgarian Academy of Sciences, 26 Acad. G. Bonchev Str., 1113 Sofia, Bulgaria

**Keywords:** rose essential oil by-products, ecofriendly products, test-systems, chromosome aberrations, micronuclei

## Abstract

The steam distillation of valuable rose essential oil from *R. damascena* Mill. and *R. alba* L. generates large volumes of wastewaters. Although such wastewaters are bio-pollutants, they contain valuable bioactive compounds. In this study we investigated the cytotoxic/genotoxic and anti-cytotoxic/anti-genotoxic potential of these products. We used cytogenetic methods for induction of chromosome aberrations and micronuclei in two different experimental test-systems: ahigher plant and human lymphocyte cultures. Different experimental schemes of treatment with the waste products showed that the genotoxic activity of wastewater from the distillation of oils from *R. alba* and *R. damascena* was low in both test–systems. Human lymphocytes showed a higher sensitivity to the products than plant cells. Both types of waste products manifested anti-genotoxic effect against N-methyl-N′-nitro-N-nitrosoguanidine, a direct mutagen. The wastewaters obtained from steam distillation of rose essential oil have cytoprotective/genoprotective effect and could decrease DNA damage. Data are promising for further use of these products in pharmacy and other areas of human life.

## 1. Introduction

Essential oil of *Rosa damascena* Mill. f. trigintipetala Dieck has brought Bulgaria world fame. The Bulgarian rose oil is of extremely high quality, due to the specific climatic conditions. Since 2014, it is in the European registry of protected geographical indications. The locality where the production of essential oils takes place in Bulgaria is the so-called ‘Valley of Roses’ near Kazanlak. Together with *Rosa damascena* Mill. there grows one more representative of the Rosaceae family—*Rosa alba* L., with delicate and fine fragrance. The two roses species used to grow together until a few years ago, so the essential oil was distilled together too. The plantations have been separated for 10 years now, and the two oils undergo distillation separately.

The rose essential oil is an attractive ingredient not only in the highest-class perfumery and cosmetics because of its unbelievable fragrance [1]. It is also of interest in pharmacy and medicine because of its valuable bioactive components. Many studies have reported that rose oil has interesting biological activities: antiviral, antibacterial, anticancer, antidepressant, antioxidant, anti-inflammatory activities. *R. damascena* essential oil also possesses relaxant, hypnotic, antisclerotic, hepatoprotective, and antispasmodic effects [2,3,4,5].

The biological activity of rose oil is due to its chemical composition. Many studies exist about the chemical content of rose essential oil of *R. damascena* from different origin [6,7,8,9,10,11] and of *R. alba* [12,13]. More than 300 different compounds are present in the rose oil from *R. damascena* [11] as well as about 214 in the rose oil from *R. alba* [12]. The main compounds are terpene alcohols citronellol, geraniol, nerol, phenethyl alcohol, saturated and unsaturated aliphatic hydrocarbons.

Industrial production of rose essential oil is mainly based on water steam distillation from fresh rose flowers followed by cohobating or concentration in which the initial distillate undergoes multiple redistillation [8]. More than 3000 kg of rose flowers produce 1 kg of rose oil by water distillation [14]. In the last 18 years the annual production of rose oil in Bulgaria has varied between 750 and 1900 kg [15].

Along with the production of the valuable rose oil, the steam distillation procedure generates two types of by-products in large amounts: wastewaters and solid residual plant materials. Discarding these wastes into the environment causes a serious problem. One kilogram of fresh raw flowers generates about 2 kg of wet waste [16]. The by-products discarded annually in the production of 1500 kg rose oil in Bulgaria amount to approximately 9,000,000 kg distilled petals [17]. In Turkey, the other main rose essential oil producer, the total amount spent flowers (distillation residues) after hydro-distillation of the fresh oil-bearing rose flowers per year is 30 thousand tonnes [18].

These waste materials are considered as bio pollutants, but at the same time they are natural products rich in biologically active compounds such as polyphenols. Researchers seek ways to utilize them and use their bioactivity in pharmacy and cosmetics. However, there are limited data on the chemical characterization of the debris, and the detailed polyphenols profile of the waste water is still unknown. Distillation extracts only the volatile constituents of the essential oil, but the non-volatile valuable components that remain in the waste are a value-added biological material.

Rusanov et al. reported some fractions of rose oil distillation wastewater (RODW) rich in phenolic compounds (ellagic acid, 2-phenylethyl-O-β-glucopyranoside, and several kaempferol and quercetin glycosides) [19]. Other authors have isolated a polyphenol-rich fraction from *R. damascena* wastewaters by Amberlite column as the stationary phase [20]. Schieber et al. obtained that kaempferol 3-O-glucoside and quercetin glycoside are the major components of flavonoids extracted from distilled rose petals of *R. damascene*,after industrial distillation for essential oil [14]. Waste rose petals of *R. damascena* also contain pectic polysaccharides [21]. The waste could be a potential source of high-value products that would have health beneficial properties [3,8,18,19,20,22,23,24,25]. Polyphenol depleted water fraction RF20-(SP-207) of rose oil distillation wastewater showed antiproliferative and antimigratory effects on Human non-tumorigenic HaCaT keratinocytes. It could serve as a basis for supportive, therapy against hyperproliferation-involved in skin diseases [26]. Polyphenol fraction from rose oil WW from *R. damascena* showed strong anti-tyrosinase activities [23].

The presence of such bioactive phytochemicals in rose waste is a potential for their application in the food industry. Antioxidant activity of rose by-products allows use as natural antioxidants in meat and sausages [27]. Extracts from rose petals manifested well-expressed antibacterial activity [3]. They could possibly find application in probiotic lactic acid bacteria dairy products [28]. Rose wastes could be additives for functionalization and aromatization of bakery products [29] and in confectionery to replace the wheat flour in pasty biscuits [30]. Mollov et al. reported that the addition of polyphenolic copigments extracted from distilled rose petals of *Rosa damascena* reduces the degradation of anthocyanins in the thermally treated strawberries beverage [31]. Some studies detected growth capabilities of young chickens depend on the intake of feed enriched with natural bioactive compounds from distilled rose petals (*Rosa damascena* Mill.) [17]. Studies have explored distilled rose waste as natural dyes, for bio-sorption of pollutants such as heavy metals, biogas production, dye-sensitized solar cells, etc. [32,33,34,35,36].

In our previous study we analyzed the chromatographic profile of the waste waters obtained from the water steam distillation of the essential oils of *R. damascena* Mill. and *R. alba* [37]. There we identified 52 chemical compounds in the waste from *R. damascena* Mill. and respectively 49 in wastewater from *R. alba L***.** The obtained data showed that 37 compounds are the same for both waste products.

The most non-volatile polyphenolic compounds, including flavonoids remain in the waste. According to [37] the total polyphenolic content in the wastewater of *R. alba* L. oil production was 7.6 ± 0.3 mg GAE/mL, and in *R. damascena*, it was 7.2 ± 0.2 mg GAE/mL, respectively. The total flavonoid content in the waste from *R. alba* L. was 1.00 ± 0.01 mg/mL and in *R. damascena* wastewater it was 1.14 ± 0.01 mg/mL, respectively. The detected flavonoids are quercetin and kaempferol, and their various derivatives, such as isoquercitrin, catechin and epicatechin. The polyphenolic acids are gallic acid, chlorogenic acid, and ellagic acid. The content of tannins in the wastewater from *R. alba* L. was 2.16 ± 0.35 mg/mL, while in the waste from *R. damascene*, it was 1.61 ± 0.05 mg/mL.

All these data call for re-evaluation of the biological properties of the by-products of hydro distillation of rose flowers. Although there are many studies addressing the valorization of the wastes and especially of the wastewaters, limited data exist about their cytotoxic/genotoxic and anti-genotoxic activities [20]. The authors investigated antioxidant activity, xanthine oxidase inhibition and a significant DNA protection ability against H_2_O_2_ of polyphenol-enriched fraction from *R. damascena* wastewaters (in concentrations of 25–100 μg/mL) on human lymphocytes.

These data, as well as the available information about the phytochemical properties of the distilled rose wastes and their well-expressed antioxidant activities, served as a background for the present study. Its aim was to investigate the cytotoxic/genotoxic and anticytotoxic/anti-genotoxic activities of concentrations of wastewaters produced after water-steam distillation of essential oils of *R. damascena* and *R. alba* in different test-systems using tests for genotoxicity. This assessment would contribute to elucidating the effectiveness and safety of these ecofriendly by-products.

## 2. Materials and Methods

### 2.1. Preparation of Rose Wastewaters

Rose wastewaters (ww)—a liquid aqueous residue used in the present study were generated because of water steam distillation done in Institute for Roses and Aromatic Plants in Kazanlak (IRAP), Bulgaria, at semi-industrial processing line.

Both roses were grown in the experimental field of the institute. The rose flowers used for distillation were from the 2019 harvest. The processing of distillation was performed at the following parameters: raw material was 8–10 kg; hydro module 1:4; the flow rate was 16–20 mL/min. The duration was 150 min. The wastewater was collected and stored at 4 °C for the next stage of the study.

### 2.2. Cytogenetic Analysis

#### 2.2.1. Chemicals

Most of the chemicals, used in the cytogenetic analysis were provided by Sigma–Aldrich Chemie GmbH, Merck (Darmstadt, Germany). N-methyl-N′-nitro-N-nitrosoguanidine (MNNG) was purchased from Fluka—AG (Buchs, Switzerland).

Two types of test-systems were used to study cytotoxic/genotoxic and anti-cytotoxic/anti-genotoxic activities of both rose wastewaters applying two of the most reliable tests for genotoxicity-induction of chromosome aberrations (CA) and induction of micronuclei (MN). Alkylating direct mutagen N-methyl -N′-nitro-N-nitrosoguanidine (MNNG) 50 μg/mL was used as a positive control for both test-systems. Untreated cells were used as a negative control.

#### 2.2.2. Plant Test-System

Seeds of the reconstructed karyotype MK 14/2034 of *Hordeum vulgare* were-presoaked for 1 h in tap water and germinated for 17 h in Petri dishes on moist filter paper at 24 °C. The barley root meristems were exposed for 1 h and/or 4 h to *R. damascene* Mill. and *R. alba* L. wastewaters at concentrations of 6, 14 and 20%. To test anti-cytotoxic/anti-genotoxic activity of rose wastewaters meristem cells were treated as follows. Part of meristems was conditioning affected (60 min) with non-toxic concentration of 20% wastewater, followed by a challenge with 50 μg/mL of MNNG (60 min) with 4 h inter-treatment time. Another part of the meristems was treated with wastewater (60 min) followed by MNNG 50 μg/mL without any inter-treatment time. After each treatment, the barley roots were washed with distilled water. After the treatment and recovery times of 18, 21, 24, 27 and 30 h, the root tips were treated for 2 h with 0.025% colchicine in a saturated a solution of α-bromonaphthalene, fixed in a solution of ethanol: acetic acid (3:1), hydrolyzed in 1 N HCl at 60 °C for 9 min, Feulgen-stained, macerated in 4% pectinase in distilled water for 12 min and at the end squashed on clean slides for scoring of metaphases with chromosome aberrations [38].

For scoring of micronuclei (MN), the barley root tips were fixed after 30 h recovery time without colchicine treatment.

#### 2.2.3. Human Lymphocytes In Vitro

Peripheral venous blood of healthy nonsmoking/nondrinking donors (men and women) aged between 33 to 40 years was used for the experiments to prepare lymphocyte cultures. All procedures were conducted corresponding to the Declaration of Helsinki and all donors signed written informed consent forms. Each lymphocyte cultures contained RPMI 1640 medium (Sigma-Aldrich, Steinheim, Germany), 12% calf serum (Sigma-Aldrich, Buchs, Switzerland), 40 mg/mL gentamycin (Sopharmacy, Sofia, Bulgaria), and 0.1% phytohemagglutinin PHA (Sigma-Aldrich, Mannheim, Germany). The experiments for chromosome aberrations were performed according to the method of Evans, [39]. The lymphocytes were treated with each rose wastewater in concentrations of 3, 6, 11, 14 and 20% for 1h and 4 h. To test anti-cytotoxic/anti-genotoxic activity of rose wastewaters some cultures were conditioning treated with wastewater (60 min) with non-toxic concentration (6%) followed by 4 h of inter-treatment time and after that challenged with 50 μg /mL of MNNG (60 min). Another part of the lymphocytes was affected with 6% of wastewater (60 min), followed immediately by MNNG 50 μg /mL (60 min) without any inter-treatment time. After each treatment, the lymphocytes were washed in fresh medium and cultured at 37 °C. At the 72nd hour of cultivation 0.02% colchicine was added to each sample, followed by 0.56 % KCl, fixation in methanol: acetic acid (3:1, *v/v*), and stained in 2% Giemsa.

For analysis of micronuclei (MN) cytochalasin-B (6 μg/mL) was added to each culture at the 44th hour after PHA stimulation to arrest cytokinesis according to cytokinesis-block micronucleus (CBMN) assay [40]. At the 24th hour after adding Cyt-B the lymphocyte cultures were centrifuged, hypotonized with 0.56% KCl and fixed in methanol: acetic acid (3:1). After centrifugation the suspension was dropped onto clean slides and stained in 2% Giemsa.

#### 2.2.4. Endpoints

**Figure 1:**
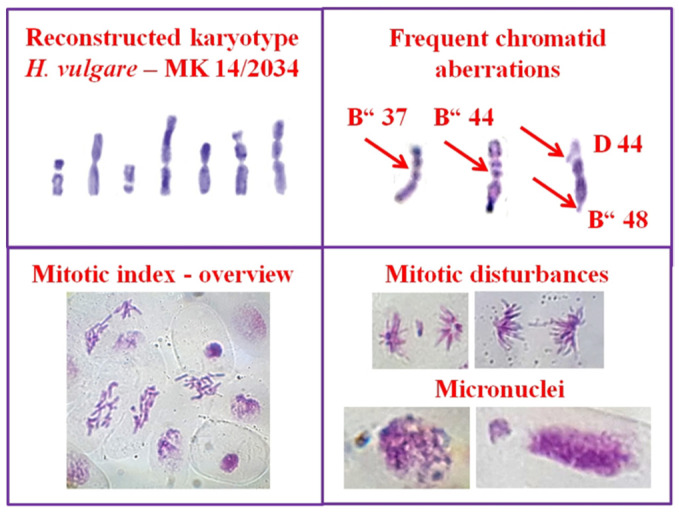
Chromosome aberrations, micronuclei, and mitotic disturbances observed in *H. vulgare* reconstructed karyotype after treatment with wastewaters produced after water steam distillation of essential oils of Bulgarian *R. damascena* and *R. alba*.

**Figure 2:**
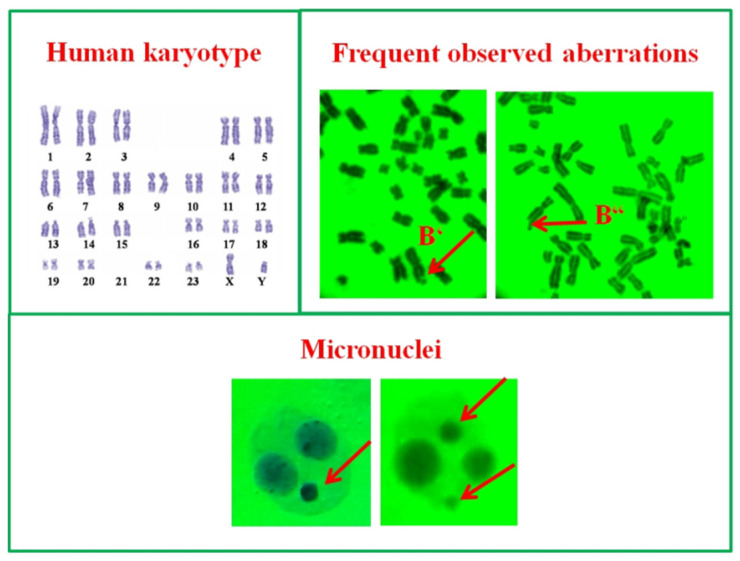
Chromosome aberrations and micronuclei observed in human lymphocyte cells after treatment with wastewaters produced following water steam distillation of essential oils of Bulgarian *R. damascena* and *R. alba*.

The percentage of metaphases with chromosome aberrations (MwA% ± SD) was calculated using test for chromosome aberrations, to assess the genotoxic effect of the wastewaters in both test systems. Chromatid, isochromatid breaks, chromatid translocations and intercalary deletions were determined (Figure 1 and Figure 2). The cell division that gives information about cytotoxicity was assessed by value of mitotic index (MI) that represents the number of metaphases per 1000 observed cells per each experimental variant. “Aberration hot spots” in barley chromosomes (reconstructed barley karyotype MK14/2034) were determined.

Using a test for micronuclei, the percentage of micronuclei (MN% ± SD) was calculated for both barley and human lymphocyte cells based on 5000 cells per each experimental variant (Figure 1 and Figure 2). In human lymphocytes, the nuclear division index (NDI) was calculated, which gives additional information about cytotoxicity of the tested substances. The following formula was used: (N1 + 2N2 + 3N3 + 4N4)/N, where N1–N4 represents the number of cells with 1–4 nuclei and N is the total number of scored cells.

#### 2.2.5. Statistics

Each experiment was repeated three times. The two-tailed Fisher’s exact test was used for statistical analysis of the different treatment variants.

## 3. Results

### 3.1. Cytotoxic and Genotoxic Effects of Wastewater from Distillation of Oils of R. alba and R. damascena

**Figure 3:**
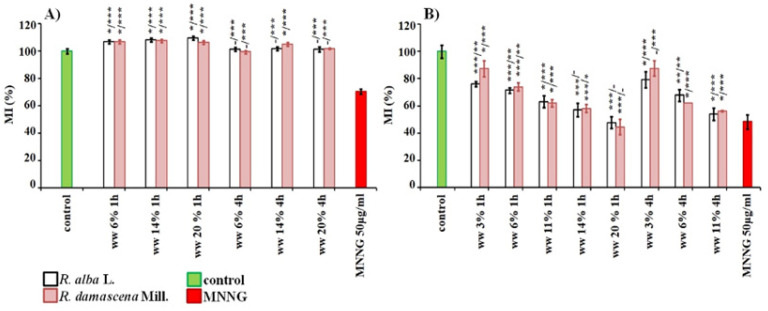
Cytotoxic activity of wastewaters (ww) obtained from distillation of essential oils from *R. alba*, and *R. damascena* assessed by the value of MI in: *H. vulgare* (**A**) and in human lymphocytes (**B**). Mitotic activity was assessed as a percent of negative control. * *p* < 0.05, ** *p* < 0.01, *** *p* < 0.001, and—non-significantly versus negative control (before slash) versus positive control MNNG (after slash).

Both wastewaters were tested for cytotoxicity using value of mitotic index (MI) as endpoint. No cytotoxic effect was observed both for wastewater from *R. alba* and *R. damascena* applied at concentrations of 6–20% for 1 and 4 h in *H. vulgare,* compared to the untreated root tip meristems (Figure 3A). Human lymphocyte cultures were more sensitive to wastes than barley. The values of mitotic index decreesed with increasing the wastewater concentration (3, 6, 11, 14 and 20%), irrespectively of the duration of treatment (Figure 3B).

**Figure 4:**
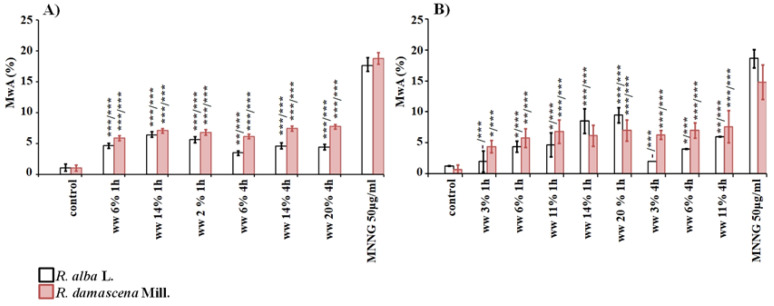
Genotoxic effect of wastewaters (ww) obtained from distillation of essential oils from *R. alba*, and *R. damascena* assessed by induction of chromosome aberrations (CA) in: *H. vulgare* (**A**) and human lymphocyte cultures (**B**). * *p* < 0.05, ** *p* <0.01, *** *p* <0.001, and—non-significantly versus negative control (before slash) versus positive control MNNG (after slash).

In genotoxic analysis conducted by induction of chromosome aberrations of variants treated with wastewater from *R. alba*, a low but statistically significant (*p* < 0.05; *p* < 0.001) clastogenic effect was found for all tested concentrations (3–20%), compared to the untreated control both in plant test-system and in human lymphocytes (Figure 4). It should be noted that lymphocyte cultures are more sensitive than barley cells to the wastewater of *R. alba*, as the frequency of chromosome damage is higher. In *H. vulgare*, no clear concentration dependence was observed for both 1 h and 4 h treatments (Figure 4A). The frequency of induced chromosome aberrations ranged from 4.7% ± 0.35 (for 6%) to 5.6% ± 0.48 (for 20%) when the cells were treated for 1 h and from 3.5% ± 0.36 (for 6%) to 4.4% ± 0.49 (for 20%) when the treatment was for 4 h. Human lymphocytes showed a clear concentration dependence with an increase in the frequency of chromosome aberrations after treatment with the waste product for 1 h and for 4 h (Figure 4B). The highest frequency of aberrations was reported in the cultures affected with a concentration of 20% (9.50% ± 0.70 for 1 h). The lowest was reported for a concentration of 3% for 1 h and 4 h (2.00% ± 1.70).

The wastewater from *R. damascena* showed a low but statistically significant clastogenic effect (*p* < 0.05; *p* < 0.001) with all tested concentrations compared to the negative, untreated control in both test-systems (Figure 4). In *H. vulgare* the frequency of aberrations after 1 h treatment was in the range of 5.9% ± 0.41 (with 6%) to 6.8% ± 0.47 (with 20%), and treatment for 4 h induced from 6.1 % ± 0.37 (with 6%) to 7.7% ± 0.38 (with 20%). No dependence on the duration of treatment and applied concentration was found with respect to the values of induced aberration in human lymphocytes, as well as in *H. vulgare* (Figure 4). The frequency of observed chromosomal abnormalities ranged from 4.40% ± 1.00 (for 3%/1 h) to 7.60% ± 2.60 (11%/4 h). The wastewater from *R. damascena* induced higher frequencies of chromosome damages than the wastewater from *R. alba* in both test-systems after treatment for 1 h and 4 h, except for the concentrations of 14% and 20% (1 h) in lymphocyte cultures (Figure 4).

The genotoxic activity of both wastewaters at all studied concentrations was much lower (*p* < 0.001) than that of the alkylating mutagen MNNG in both plant and lymphocyte test- systems (Figure 4).

**Figure 5:**
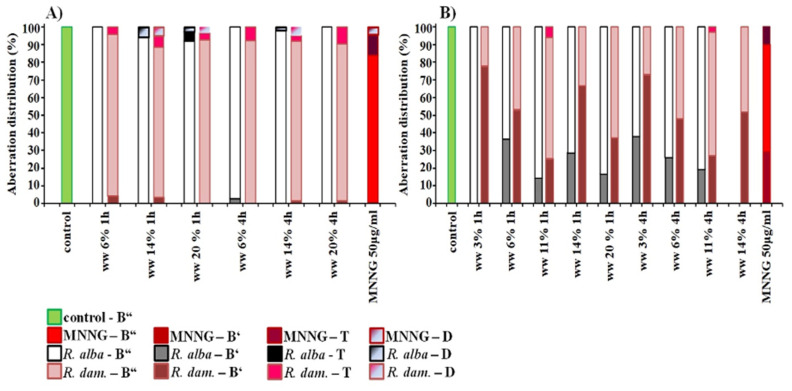
Distribution of aberrations observed after treatment with wastewaters (ww) of *R. alba* and *R. damascena* in *H. vulgare* (**A**) and human lymphocytes (**B**), B”—isochromatid breaks, B’—chromatid breaks, T—translocations, D—intercalary deletions.

The spectrum of observed chromosome aberrations in *H. vulgare* induced by different concentrations of wastewater of *R. alba*, is composed mainly of isochromatid breaks and a small number of chromatid breaks. In human lymphocyte cultures predominantly isochromatid breaks followed by chromatid breaks, with no structural chromosomal changes such as translocations were observed (Figure 5).

Mainly isochromatid breaks followed by translocations, intercalary deletions and chromatid cleavages in *H. vulgare* were induced by wastewater from *R. damascena* in *H. vulgare*. More chromatid breaks were detected in human lymphocytes in variants treated with *R. damascena* waste compared to that of with *R. alba* wastewater. A small percentage of translocations were also observed.

The aberration hot spots in *H. vulgare* in reconstructed karyotype showed that the wastewater from *R. alba* has the potential to reduce their occurrence. In the variants with conditioning treatments with 20% wastewater, aberration hot spots decreased by 60% compared to those induced by the MNNG treatment alone (Table 1).

**Figure 6:**
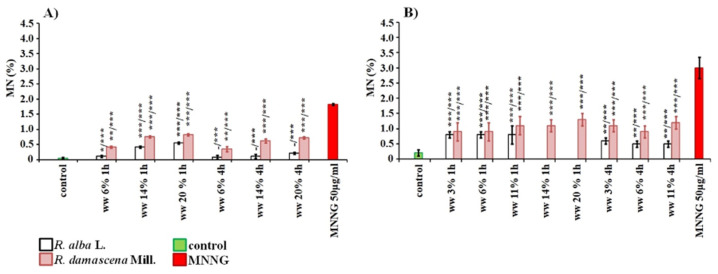
Genotoxic effect of wastewaters (ww) obtained from distillation of essential oil from *R. alba* and *R. damascena* assessed by the induction of micronuclei (MN) in: *H. vulgare* (**A**) and human lymphocyte cultures (**B**). * *p* < 0.05, ** *p* <0.01, *** *p* < 0.001, and—non-significantly versus negative control (before slash), versus positive control MNNG (after slash).

The cytotoxic and genotoxic effects of both types of wastewaters were assessed with one more endpoint for genotoxicity—induction of micronuclei (MN) (Figure 6). *H. vulgare* meristem cells were treated with concentrations from 6 to 20% and human lymphocytes treated for concentrations from 3 to 11% for 1 h and 4 h (*R. alba*) and from 3% to 20% with *R. damascena*. A low but significant increase in the value of micronuclei induced by waste of *R. alba* while increasing the concentration was found in barley compared with the negative control. The value of the induced MN varied from 0.10% ± 0.01 (6%) to 0.23 ± 0.06 (20%) in the case of 1 h treatment and from 0.13% ± 0.06 (6%) to 0.57% ± 0.06 (20%) in the case of the longer period of treatment—4 h (Figure 6A). In lymphocyte cultures, all wastewater concentrations of *R. alba* induced close values of micronuclei (0.80% ± 0.10 at 3%, 6 % and 11% for 1 h and 0.50% ± 0.10 at 6% and 11% for 4 h), which is higher (*p* < 0.001) than that of the negative control 0.20% ± 0.10 (Figure 6B). It is interestingly to note, that with the extension of the treatment time to 4 h, the values of the induced micronuclei were lower than those observed after 1 h, probably due to the induction of defense mechanisms in the cells.

The observed micronuclei after treatment with *R. damascena* wastewater ranged from 0.90% ± 0.30 (after treatment with 3%/1 h) to 1.30% ± 0.20 (with 20%/1 h) in lymphocyte cultures (Figure 6B). No concentration dependence and treatment duration dependence were observed after treatment with the waste product of *R. damascena* essential oil distillation. In barley the lowest concentration of 6% induced the lowest number MN, whereas close micronucleus frequencies were reported for 14 and 20%, and no significant difference was found between them.

The genotoxicity of both wastewaters applied in all used by us concentrations was much lower (*p* < 0.001) than that of the positive control MNNG in plant as well in lymphocyte test-system (Figure 6).

**Figure 7:**
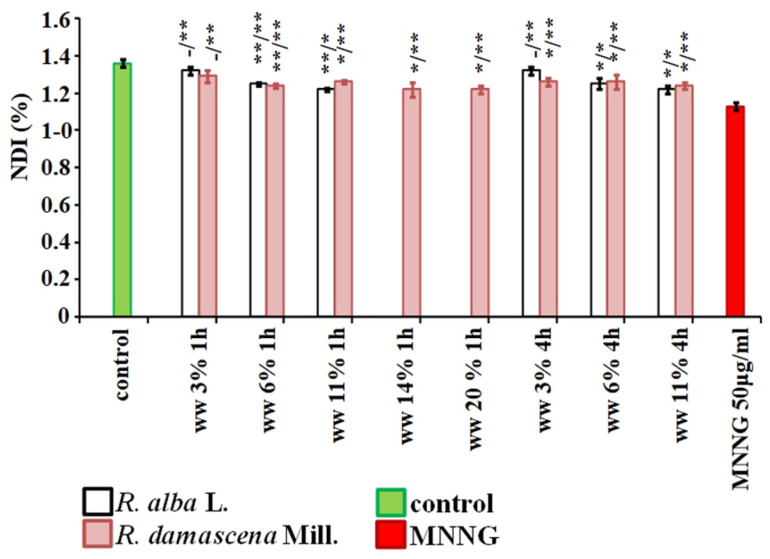
Cytotoxic potential of wastewaters (ww) obtained from distillation of essential oil from *R. alba* and *R. damascena,* assessed by the value nuclear division index (NDI) in human lymphocyte cultures after application of different schemes of treatment. * *p* < 0.05, ** *p* < 0.01, and—non-significantly versus negative control (before slash), versus positive control MNNG (after slash).

Nuclear division index (NDI), used as another indicator for assessment of the cytotoxic activity of the wastewaters, was calculated for lymphocyte cultures. Its value was reduced (*p* < 0.01) by treatment with the higher concentrations of wastewater from *R. alba* 6% and 11% compared to the negative control. Calculating the NDI in the variants treated with wastewater from *R. damascena*, a slight, but in some treatment variants a significant decrease in the index was found compared to the negative control (1.35% ± 0.02) (Figure 7). It ranged from 1.29% ± 0.03 (for 3.03%/1 h) to 1.22% ± 0.04 (for 14%, 20% /1 h and for 14%/4 h).

### 3.2. Anti-Cytotoxic and Anti-Genotoxic Activities of Wastewaters from Distillation of Oils of R. alba and R. damascena

**Figure 8:**
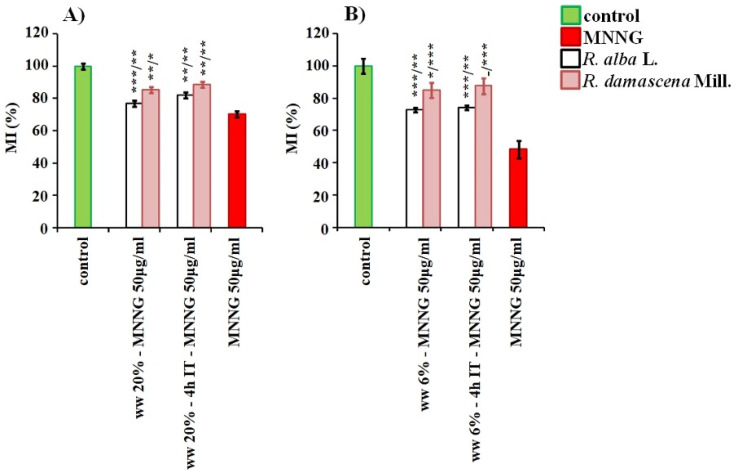
Anti-cytotoxic activity of wastewater (ww) from *R. alba*, and *R. damascena* assessed by the value of mitotic index (MI) after application of different experimental schemes of treatment with wastewater conditioning prior to MNNG challenge (50 μg/mL) with 4 h inter-treatment time and without any inter-treatment time in: *H. vulgare* (**A**) and in human lymphocytes (**B**). Mitotic activity was assessed as a percent of negative control. * *p* < 0.05, ** *p* < 0.01, *** *p* < 0.001, and—non-significantly versus negative control (before slash), versus positive control MNNG (after slash).

The waste products of *R. alba* demonstrate a well pronounced anti-cytotoxic potential (*p* < 0.001, *p* < 0.01) against direct mutagen MNNG when the schemes with combined treatment was applied in both test-systems. This was obtained as in variants with conditioning treatment with concentration of 20% (for barley) and with 6% (for human lymphocytes), followed by a challenge with MNNG (50 μg/mL) and 4 h inter-treatment time, as well as in variants without any time between treatments (Figure 8). The values of MI in these combined variants were higher than in the variants with the mutagen, and reach the values calculated for samples with the wastewater only.

The similar anti-cytotoxic effect was detected also for wastewater of *R. damascena* assessed by MI applying the same experimental schemes with combined treatment as that with waste of *R. alba* (Figure 8). The cytotoxic activity was significantly lower (*p* < 0.001) compared with that of direct mutagen in both plant and lymphocyte test-systems both in variants with conditioning treatment with non-toxic concentrations of wastewater from *R. damascena* (20% for barley and 6% for human lymphocytes, respectively), followed by the challenge with MNNG (50 μg/ml) with 4 h inter-treatment time, and without any inter-treatment time.

**Figure 9:**
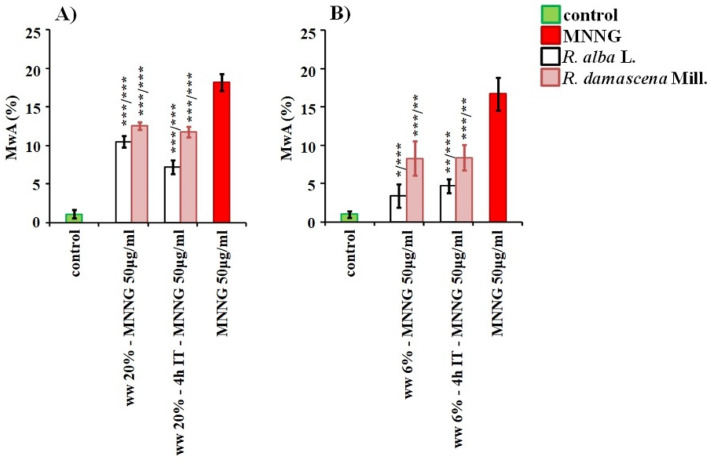
Anti-genotoxic effect of wastewaters (ww) from *R. alba*, and *R. damascena* assessed by induction of chromosome aberrations (CA) after application of different experimental schemes of treatment with: -wastewater conditioning prior to MNNG challenge (50 μg/mL) with 4 h inter-treatment time and—without any inter-treatment time in: *H. vulgare* (**A**) and in human lymphocytes (**B**). * *p* < 0.05, ** *p* < 0.01, *** *p* < 0.001, and—non-significantly versus negative control (before slash), versus positive control MNNG (after slash).

Assessing the anti-genotoxic potential of wastewater from *R. alba* by endpoint for genotoxicity CA, applying both schemes with combined treatments with non-toxic concentrations of wastewater and harmful effects of MNNG in both test-systems, a clear anti-genotoxic effect was observed (Figure 9).

A significant decrease (*p* < 0.001) of the frequency of induced chromosome aberrations (4.70% ± 0.90 and 3.40% ± 1.50 in lymphocytes and 7.2% ± 0.89 in barley) compared to those observed after treatment with MNNG alone (50 μg/mL) in barley (17.6% ± 1.28) and lymphocyte cultures (18.60% ± 1.50), was obtained. The values of aberrations in barley were twice as low and in lymphocyte cultures—five times as low (Figure 9). No significant difference was obtained between the frequencies of aberrations induced after different variants of combined treatment in human lymphocytes, whereas in *H. vulgare* the conditioning treatment with waste from *R. alba* followed by challenge with mutagen and 4 h inter-treatment time reduced the chromosome aberrations to a higher extent (ww *R. alba*→ MNNG10.5% ± 0.72 versus ww *R. alba*→ 4 h → MNNG7.2% ± 0.89 which indicates 31% stronger protection against MNNG).

Wastewater from *R.damascena* also exhibited anti-clastogenic activity after application of both schemes with combined treatment, with 4 h inter-treatment time and without any inter-treatment time. The frequencies of chromosome aberrations were decreased (*p* < 0.01, *p* < 0.001) compared to those of the alkylating agent MNNG in cells of both test-systems (Figure 9). Close values of aberrations were obtained for both variants with combined treatment in barley as well in human lymphocytes.

As seen from Figure 9, the waste water from *R. alba* has more pronounced defense potential against MNNG than waste water from *R. damascena*, as the frequency of chromosome aberrations was decreased to a higher extent for both test-systems (*p* < 0.001).

**Figure 10:**
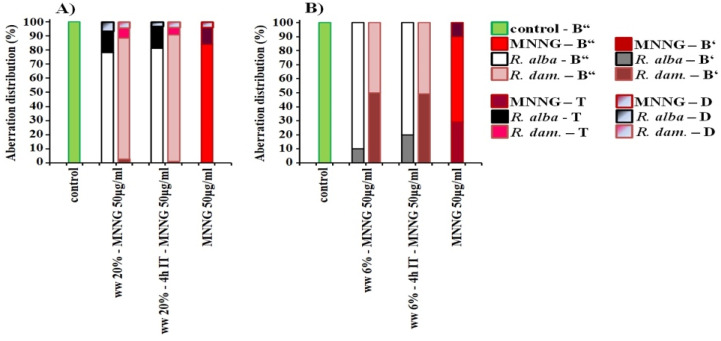
Distribution of aberrations observed after different experimental schemes of treatment with rose wastewaters (ww) and MNNG (50 μg/mL) in: *H. vulgare* (**A**) and in human lymphocytes (**B**), B”—isochromatid breaks, B’—chromatid breaks, T—translocations, D—intercalary deletions.

Analyzing the spectrum of induced chromosome aberrations in the variants with combined treatment with waste from *R. alba* and MNNG in barley, along with the isochromatid breaks and a low number of chromatid breaks, translocations and intercalary deletions was also detected (Figure 10). In human lymphocytes mainly isochromatid breaks followed by chromatid breaks were observed. The spectrum of aberrations observed after combined treatment with wastewater from *R. damascena* essential oil production and MNNG in *H. vulgare* was consist mainly of isochromatid breaks followed by translocations, intercalary deletions and chromatid breaks whereas in human lymphocytes the chromatid breaks were more than that in variants with *R. alba* wastewater.

Analysis of the aberration hot spots in *H. vulgare* meristem cells treated with non-toxic conditioning treatment with 20% wastewater from *R. alba* showed that they decrease by 60% compared to those induced by MNNG alone. Aberration hot spots detected after conditioning treatment with 20% waste product from *R. damascena* and subsequently affected with MNNG were 33% less compared with those induced with MNNG alone and 67% less when the experimental scheme of treatment was wastewater 20% → 4 h → MNNG (Table 2).

**Figure 11:**
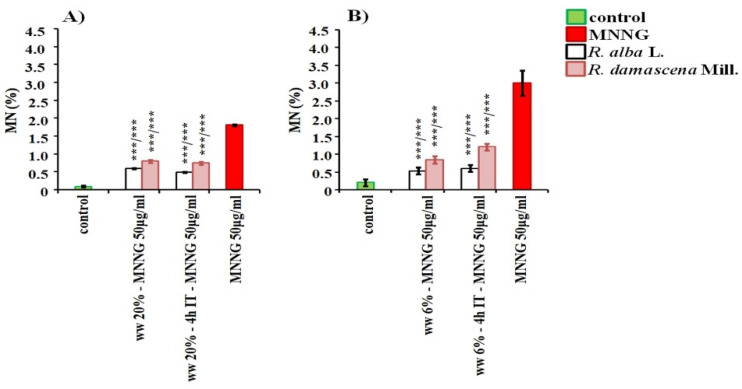
Anti-genotoxic effect of wastewaters (ww) from *R. alba*, and *R. damascena* assessed by induction of micronuclei (MN) after application of different experimental schemes of treatment with: -wastewater conditioning prior to MNNG challenge (50 μg/mL) with 4 h inter-treatment time and,—without any inter-treatment time in: *H. vulgare* (**A**) and in human lymphocytes (**B**). *** *p* < 0.001, and non-significantly versus negative control (before slash) versus positive control MNNG (after slash).

Well-defined anti-genotoxic potential (*p* < 0.001) of the wastewater from *R. alba*, was observed also using MN as endpoint for genotoxicity, applying the experimental schemes with combined treatment in both test-systems. The induction of MN was decreased more than five times in lymphocyte cells and twice in barley compared to those of the alkylating direct mutagen (Figure 11). The wastewater from *R. damascena* also exhibits a very pronounced defense potential against damages induced by alkylating direct mutagen MNNG assessed by MN induction. A significant decrease of the frequencies of MN (*p* < 0.001) compared with that induced by MNNG (2.50% ± 0.30) was obtained using the experimental schemes with combined treatment (with time between treatments and without any) both in the plant test system and in the lymphocyte cultures. The frequency of MN observed in lymphocyte cultures after these treatments was even lower (0.84% ± 0.11) than that of the wastewater alone (0.90% ± 0.30).

**Figure 12:**
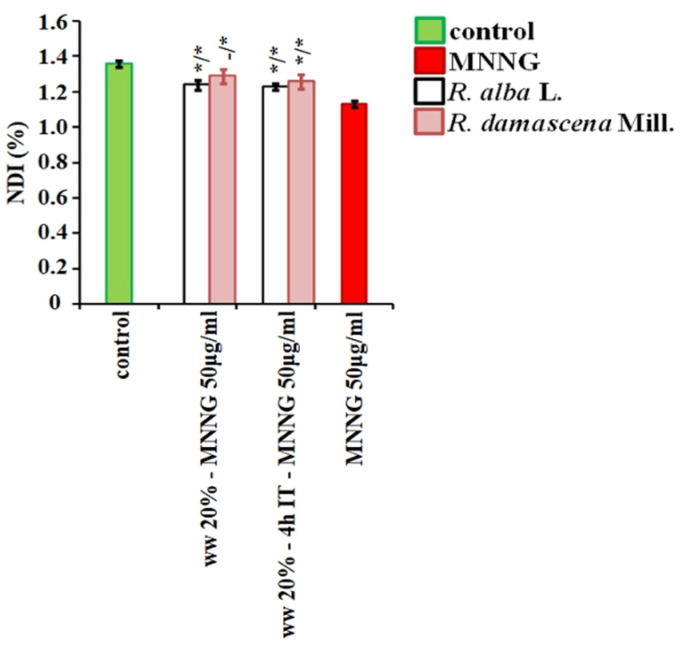
Anti-cytotoxic activity of wastewater (ww) from *R. alba*, and *R. damascena* assessed by the value of nuclear division index (NDI) in human lymphocyte cultures after application of different schemes of treatment. * *p* < 0.05, and non-significantly versus negative control (before slash), versus MNNG (after slash).

NDI calculated for variants where the experimental schemes with conditioning treatment with wastewater of *R. alba* with 4-h inter-treatment time between treatments and without any time were applied, was significantly higher compared to that of the positive MNNG control (Figure 12). The variants with combined treatment with waste from *R. damascena* had higher level of NDI 1.29% ± 0.04 compared with that of MNNG and close to that of the single treatment with wastewater. This data support the results obtained both with MN and CA endpoints.

## 4. Discussion

**Figure 13:**
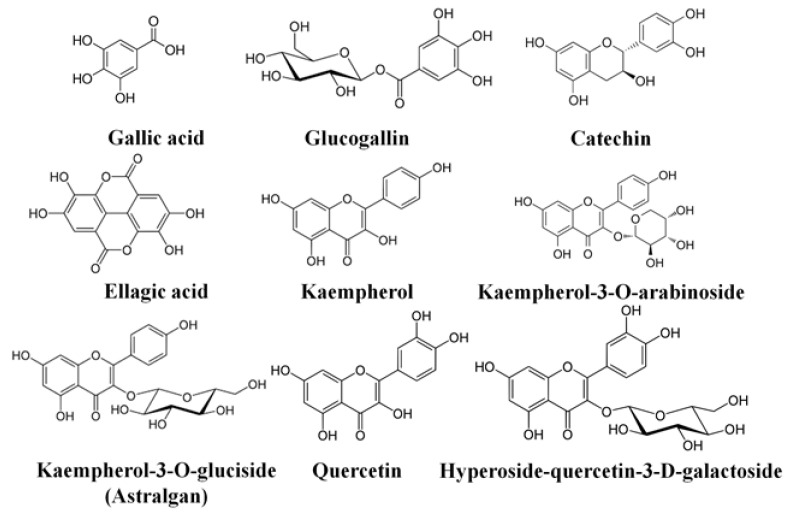
Basic chemical constituents in wastewater from *R. damascena*, and *R. alba* by UHPLC-HRMS/MS analysis (according to Georgieva et al. [37]).

Rose oil is an economically important product for Bulgaria. Along with the valuable essential oil, however, the production generates many waste products that pollute the environment. There has been a growing interest in the study of their utilization in recent years The main components of the rose wastewaters are polyphenols and polysaccharides. The by-product from *R. damascena* oil distillation contains kaempferol, quercetin, ellagic acid and their glycoside derivatives [19,22,23]. The chromatographic analysis of the present wastewater from water steam distillation of essential oils of Bulgarian *R. damascena* and *R. alba* [37], detected various mono-, di-, and acylated glycosides of polyphenolic compounds quercetin and kaempferol, ellagic acid, as well as many of their derivatives, gallic acid and its derivatives—catechin and epicatechin. (Figure 13).

Their quantity and quality varied depending on the rose species that generated the wastewater. Gallic acid is about two times more in *R. alba* L. than in *R. damascena*; ellagic acid is also present in a larger amount in *R. alba* than in *R. damascena.* Hyperoside and isoquercitrin are present more than twice higher in *R. damascena* than in *R. alba* The quantities of kaempherol-3-O-glucoside, kaempherol-3-O-arabinoside and kaempherol-p-coumarouyl-hexoside are more than 2.5 times higher in *R. damascena* than in *R. alba* [37]. This suggests some differences in the biological activity of the wastewater. Hence, it was interesting to investigate and compare the cytotoxic/genotoxic activities and anti-cytotoxic/anti-genotoxic potential of concentrations of wastewater derived from the production of essential oils of both rose species, *R. damascena* and *R. alba* using two different types of experimental test-systems.

At concentrations from 6% to 20% both types of wastewaters did not induce any cytotoxic effect in *H. vulgare* test-system. The human lymphocytes were more sensitive than barley and the cell’s viability and mitotic activity clearly depended on the concentration (3% to 20%) and treatment time duration (1 h and 4 h). This was so both in the MI and NDI assay. Wedler et al. [26] also reported a dose dependent antiproliferative activity of rose wastewater fraction in non-tumorigenic HaCaT cells. Some authors reported that a fraction of wastewater from *R. damascena* is toxic at concentrations of 100 μg/mL and higher in HepG2 cells in the MTT assay [20]. Georgieva et al. have found that wastewater from *R. damascena* Mill. and of *R. alba* L. did not exert very strong cytotoxic effect on some human cancer and normal cell lines in the MTT assay, but different type of cells showed different sensitivity to the tested substances [37]. The cells differ in their sensitivity to the tested substances.

In our study both kinds of rose wastewater applied for 1 h and 4 h at concentrations in the range of 3–20% demonstrated clastogenic activity to DNA in both test-systems compared to the negative control. The hereditary material of the human lymphocytes was more susceptible to damage than that of barley. Conversely, in a study by other authors [20], treatment for 15 min with wastewater fraction from *R. damascena* (25, 50, 100 μg/mL) did not show any genotoxic effect in the comet assay in human lymphocytes. This was probably because of the shorter treatment time duration in [20], as both lower concentrations are similar to those used by us.

Here, we established that the value of induced chromosome aberrations depends on the type of the wastewater. *R. alba* wastewater showed lower clastogenic activity than that of *R. damascena* in both test-systems. The frequency of damage was lower after treatment for a longer time of 4 h than for 1 h. The frequency of chromosome aberrations induced by the by-products from *R. damascena* was not time-dependent. Interestingly, the treatment with waste from *R. alba* essential oil distillation induced predominantly chromatid breaks and less isochromatid breaks in lymphocyte cells, whereas both types of breaks were with almost equal values after treatment with waste from *R. damascena* These results probably reflect some differences in the chemical composition and the quantitative ratio of the components in the two kinds of wastewater. More substances were found in the waste of *R. damascena* than in that of *R. alba*. The observed cytotoxic and genotoxic effects of the wastes is probably due on the presence of quercetin and kaempferol. The information available in the literature reports such activities of quercetin [41] and kaempherol [42] and weaker ones of ellagic acid [41] in various cancer cells. These effects depend on a complex of conditions. The concentrations used, target organisms or cell lines are important [43]. The treatment scheme, including possible combinations with other drugs, is also of great importance.

We tested the anti-cytotoxic and anti-genotoxic activities of both wastewaters against the direct mutagen MNNG by applying two experimental schemes using tests for genotoxicity. Our results showed that both kinds of wastewater possessed anti-cytotoxic and anti-genotoxic potential. The frequencies of chromosome aberrations and micronuclei decreased following conditioning treatment with the wastewater in a non-toxic concentration before MNNG challenge with 4 h inter-treatment time, compared to the samples treated only with MNNG. The defense potential of the waste products manifests itself independently of the experimental conditions in both test-system. The damage induced by the alkylating agent also decreased both in barley and in lymphocytes when non-toxic concentrations (6% for lymphocytes and 20% for barley) of rose wastewater were applied before the mutagen without any inter-treatment time. The protective effect demonstrated by both kinds of wastewater in our study is probably due to the presence of polyphenolic compounds, which are the major components of both kinds of rose water by-products. Our results are in accordance with the study of Georgieva et al. [37], who obtained a well-expressed redox-modulating capacity of wastewater from four Bulgarian rose species including *R. alba* and *R. damascena* The fact that the defense potential of the waste from *R. damascena* manifested itself to a lesser extent than that of *R. alba* is probably due to a difference between the chemical composition of the wastewater. Georgieva et al. [37] showed a slightly higher value of total polyphenolic content in the waste of *R. damascena* than in *R. alba*. The chromatographic profile previously reported [37] showed that both kinds of wastewater contain hyperoside, mono-, di-, and acylated glycosides of kaempherol and quercitin and their derivatives, as well as ellagic acid and gallic acid and their derivatives. These compounds are known for their biological activities- antioxidant, antiradical activities, and other pharmacological properties [44,45,46,47]. The presence of these main chemical compounds in the tested by-products would explain the defence potential in our study. The flavonoid kaempferol can protect DNA, proteins and lipids against damage induced by oxidative stress. It has manifested anticancer and anti-inflammatory activities [48,49]. Our previous study showed its anti-cytotoxic and anti-genotoxic potential against the radiomimetic zeocin [50]. The kaempferol glycoside derivatives can decrease DNA damage induced by etoposide in human peripheral blood mononuclear cells [51]. Ellagic acid has anti-genotoxic potential in human sperm [52] and in Zebrafish Blood Cells exposed to benzene [53], It also has anticarcinogenic and hepatoprotective properties [45,54]. Both ellagic acid and quercetin significantly increase the value of GSH and decrease NADPH and ascorbate dependent lipid peroxidation in mice [55]. Quercetin is well known by its anticancerogenic effect [41,56]. Quercetin has anti-genotoxic activity against DNA damage induced by the mutagen methyl methane sulfonate, aflatoxin B1, and doxorubicin in human hepatoma HepG2 cells [44]. The authors suggest that quercetin acts both as a desmutagenic and bioantimutagenic agent, repairing DNA damage. Tannins, which are secondary metabolites derived from phenolic acids also show antimutagenic effects in terms of DNA-breaking activity [57] and possess anticancerogenic activity [58].

The well-known monofunctional alkylating mutagen N-methyl-N’-nitro-N-nitrosoguanidine used in the present study, directly alkylates the nitrogen and oxygen end of the DNA bases and can induce intra-strand, inter-strand crosslinks and double-strand breaks in DNA [59]. Base excision reparation(BER) and nucleotide excision reparation (NER) are the main processes for the repair of N-alkylated bases [60,61]. The well expressed anti-cytotoxic and anti-genotoxic effects of the wastewater from water steam distillation of rose oil from Bulgarian *R. damascena* and *R. alba* against MNNG, in our study, suggest that the chemical compounds in the waste could also activate repair functions. Such repair pathways could act in addition to the anti-oxidant and scavenging activities, of the main polyphenolic compounds. Our previous study showed that *R. alba* essential oil demonstrates good anti-cytotoxic and anti-genotoxic potential against MNNG in plant and lymphocyte test-systems [13]. Based on our present results, we suggest that the tested rose wastewater shows good defense potential and is comparable to that of fresh rose oil samples. However, more studies are necessary to confirm this.

## 5. Conclusions

This study assessed the cytotoxic/genotoxic and anti-cytotoxic/anti-genotoxic effects of wastewater produced after water steam distillation of essential oils of Bulgarian *R. damascena* and *R. alba*. The waste products did not show strong cytotoxic/genotoxic effects. This depends on the type of rose (their polyphenolic content) from which the waste originated, the concentrations applied and the sensitivity of the test-system used. Both kinds of rose wastewater showed good biological effect, i.e., cytoprotective/genoprotective activity against the direct alkylating mutagen MNNG. The level of DNA damage decreased, regardless of the experimental conditions in two different types of test-systems. The obtained data are promising for further utilization and successful use of these by-products in appropriate concentrations in pharmacy and other areas of human life, just as the rose essential oils themselves. On the other hand, the valorisation of these products would contribute to the reduction of waste from rose oils distillation in the environment.

## Figures and Tables

**Table 1:** Aberration “hot spots” observed in the chromosomes of root meristem cells of barley—the reconstructed karyotype MK14/2034 after different experimental schemes of treatment with wastewater from the production of essential oil from *R. alba* and *R. damascena* and MNNG.

	Treatment Variants			Hot	Spot	Segments		
		Non-Hot Spot Segments	Chr.2 Segm.12 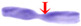	Chr.4 ^3^ * Segm.21 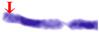	Chr.6 Segm.37 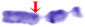	Chr.6 Segm.39 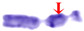	Chr.7 ^1^ * Segm.44 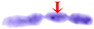	Chr.7 ^1^ * Segm.48 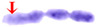
**1**	Untreated control	100%	-	-	-	-	-	-
**2**	*R. alba* ww 6% 1 h	100%	-	-	-	-	-	-
	*R. damascena* ww 6% 1 h	85.7%	-	-	-	-	-	14.3%
**3**	*R. alba* ww 14% 1 h	100%	-	-	-	-	-	-
	*R. damascena* ww 14% 1 h	100%	-	-	-	-	-	-
**4**	*R. alba* ww 20% 1 h	73.7%	-	-	15.8%	10.5%	-	-
	*R. damascena* ww 20% 1 h	87.3%	-	-	-	-	-	12.7%
**5**	*R. alba* ww 6% 4 h	100%	-	-	-	-	-	-
	*R. damascena* ww 6% 4 h	100%	-	-	-	-	-	-
**6**	*R. alba* ww 14% 4 h	100%	-	-	-	-	-	-
	*R. damascena* ww 14% 4 h	87.1%	-	-	-	-	-	12.9%
**7**	*R. alba* ww 20% 4 h	86.4%	-	-	13.6%	-	-	-
	*R. damascena* ww 20% 4 h	100%	-	-	-	-	-	-
**8**	MNNG 50 µg/mL	52.2%	6.8%	5.3%	7.8%	7.1%	9.9%	10.6%

* Reconstructed karyotype of *Hordeum vulgare* (MK 14/2034) is a result of the combination of two simple reciprocal translocations between parts of chromosomes 1 and 7 and chromosomes 3 and 4.

**Table 2:** Aberration “hot spots” observed in the chromosomes of root meristem cells of barley—the reconstructed karyotype MK14/2034 after different variants of conditioning treatment with wastewater from the production of essential oil from *R. alba* and *R. damascena*.

	Treatment variants			Hot	Spot	Segments			
		Non-hot spot segments	Chr.2 segm.12 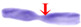	Chr.4^3^ * segm.21 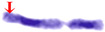	Chr.4^3^ * segm.24 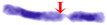	Chr.6 segm.37 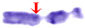	Chr.6 segm.39 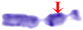	Chr.7^1^ * segm.44 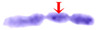	Chr.7^1^ * segm.48 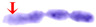
1	Untreated control	100%	-	-	-	-	-	-	-
2	*R. alba* ww 20%-MNNG50	80.4%	-	-	7.6%	-	-	12.0%	-
	*R. damacsena* ww 20%-MNNG50	77.2%	-	-	-	-	-	12.3%	10.5%
3	*R. alba* ww 20%→4 h→MNNG50	77.1%	-	-	10.9%	-	-	12.0%	-
	*R. damascena* ww 20%→4 h→MNNG50	86.0%	-	6.5%	-	-	-	-	7.5%
4	MNNG 50 µg/ml	52.2%	6.8%	5.3%	-	7.8%	7.1%	9.9%	10.6%

* Reconstructed karyotype of *Hordeum vulgare* (MK 14/2034) is a result of the combination of two simple reciprocal translocations between parts of chromosomes 1 and 7 and chromosomes 3 and 4.

## Data Availability

All the obtained data of this research are presented in the manuscript.
